# Building Climate-Resilient Health Systems in India: A Comprehensive Health Systems Approach

**DOI:** 10.7759/cureus.68951

**Published:** 2024-09-08

**Authors:** Sanjeev Kumar, Anand Kumar, Malkit Singh

**Affiliations:** 1 Public Health, Private Practice (Health System), New Delhi, IND; 2 Public Health, Private Practice (Health System), Patna, IND; 3 Medical Microbiology, Postgraduate Institute of Medical Education and Research, Chandigarh, IND

**Keywords:** building blocks, climate resilient health system, health system, india, low carbon

## Abstract

Climate change has a significant impact on human health, leading to increased mortality and morbidity worldwide, including in India. To address this issue, countries must work on developing health systems that can effectively respond, manage, recover, and adapt to climate-related shocks and stresses. Strengthening the health system's resilience requires focusing on essential building blocks, such as leadership, health information systems, the health workforce, essential medical products and technologies, service delivery, and health financing. India, as a key player in this global effort, has undertaken many initiatives in each of the health system building blocks. However, there is a pressing need for India to strengthen its planning processes, ensure adequate financial resources, and develop a robust data system to make its health system resilient to climate change and low carbon emissions.

## Editorial

Climate change, directly and indirectly, affects human health through weather patterns, such as heat and cold waves, storms, droughts, floods, etc., and indirectly by affecting water, air, and food quality [1]. WHO forecasts a global increase of about five million deaths between 2030 and 2050 because of climate change [2]. The Climate Impact Lab Study (2019) estimates that India will experience 10% of additional deaths due to climate change by the end of this century [3]. Regarding the economic losses, the Reserve Bank of India has estimated that the Indian economy could be at risk of losing 4.5% of its Gross Domestic Product (GDP) by 2030 owing to lost labor hours due to climate change, precisely due to extreme heat and humidity [4].

While the health system should respond to the healthcare needs of people affected by climate change, it also faces its capacity limitations. Health system resilience is crucial, defined as its ability to predict, prevent, prepare for, absorb, adapt, and transform during shocks and stresses while providing continuous routine health services [5]. A climate-resilient health system is expected to respond to, manage with, recover from, and adapt to climate-related stresses and shocks, fostering sustained improvements in population health despite an unstable climate [6]. Such sustainable health systems provide an opportunity for long-term human development by addressing the health impacts of climate change and promoting overall well-being. Strengthening the health system's building blocks in unstable climates is essential to safeguard population health at large.

Climate resilience involves strengthening the healthcare system to better adapt to evolving health threats due to climate variations. The health system building blocks serve as the basis for expanding key components that specifically bolster climate resilience to ensure that the enhancement of climate resilience leverages and fortifies health systems.

**Figure 1:**
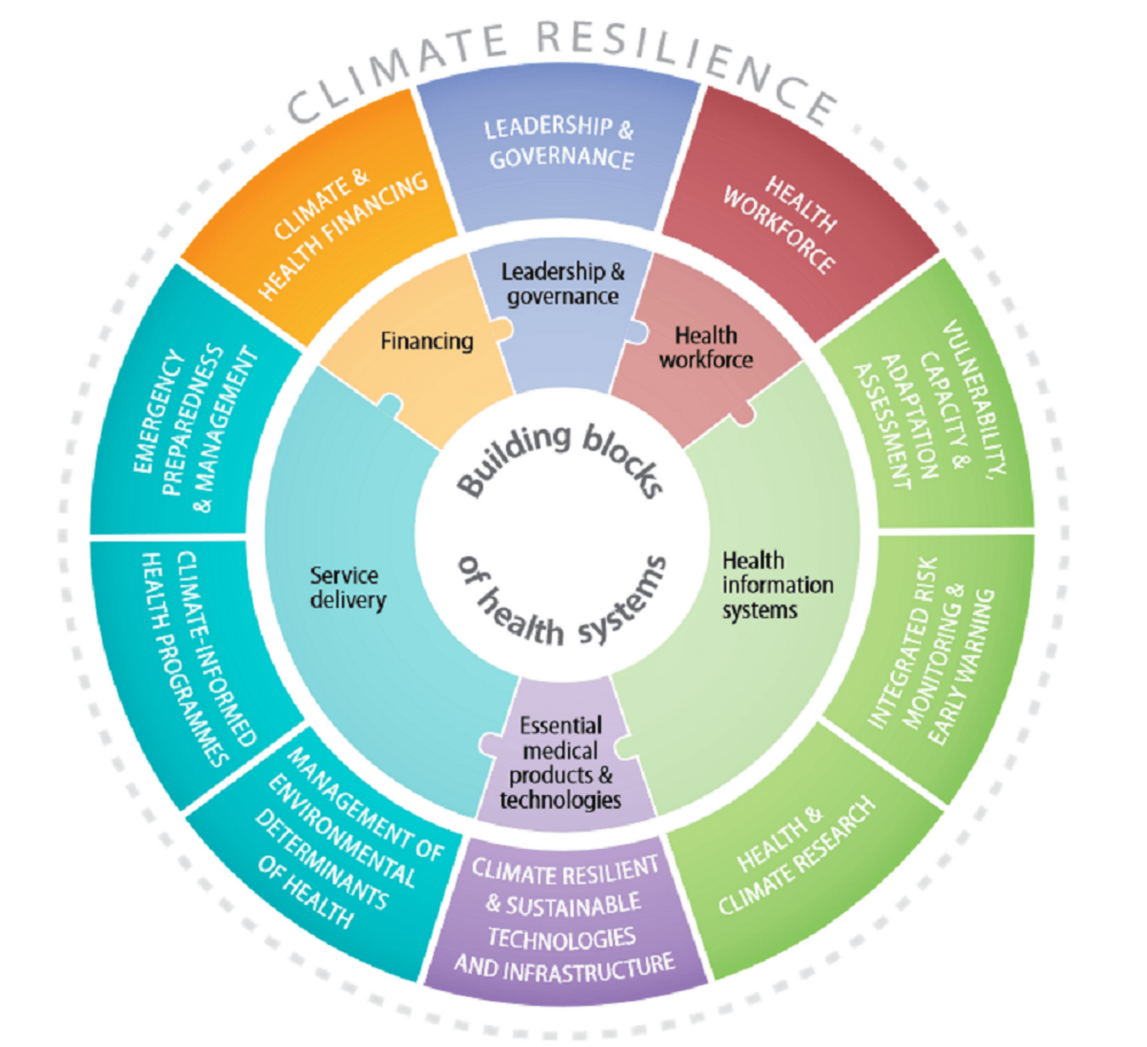
WHO operational framework for building climate-resilient health systems.

Climate change is the most significant health hazard confronting humanity; governments across the globe, including the Indian Government at union and states, are working towards improving the resilience of health systems to climate change [7]. WHO has developed the operational framework for developing a resilient health system for climate, comprising 10 components; the foundational components are leadership and governance, information systems, health workforce, essential medical products, technologies, service delivery, and health financing (Figure 1) [6]. As the health system building blocks are the primary component for achieving universal health coverage and health outcomes, we discussed its importance and initiatives taken under these building blocks at the union and state levels in India to better understand the preparedness of the Indian health system resilience for climate change. Now, all the above-mentioned components are discussed below one by one in detail.

Leadership and governance

Effective governance, evidence-based policy-making, strategic planning, and collaboration with various stakeholders are crucial for creating a health system resilient to climate change. Leadership interventions are necessary to manage the shock of the health system due to climate variations and chart the strategic health policies. Prioritizing health in national climate change policy and integrating climate change considerations into health policies and programs are vital. Political leadership is needed to address health risks related to climate change in other sectors' programs. Greater collaboration between the health sector and other sectors influencing health, such as agriculture and urban planning, is required to tackle the root causes of climate change and its health impacts. Establishing a “climate change and health” department within the Ministry of Health is essential for planning and managing climate-resilient programs, fostering inter-sectoral collaboration, formulating and implementing national health and climate change strategies, and developing agreements with key stakeholders.

**Figure 2:**
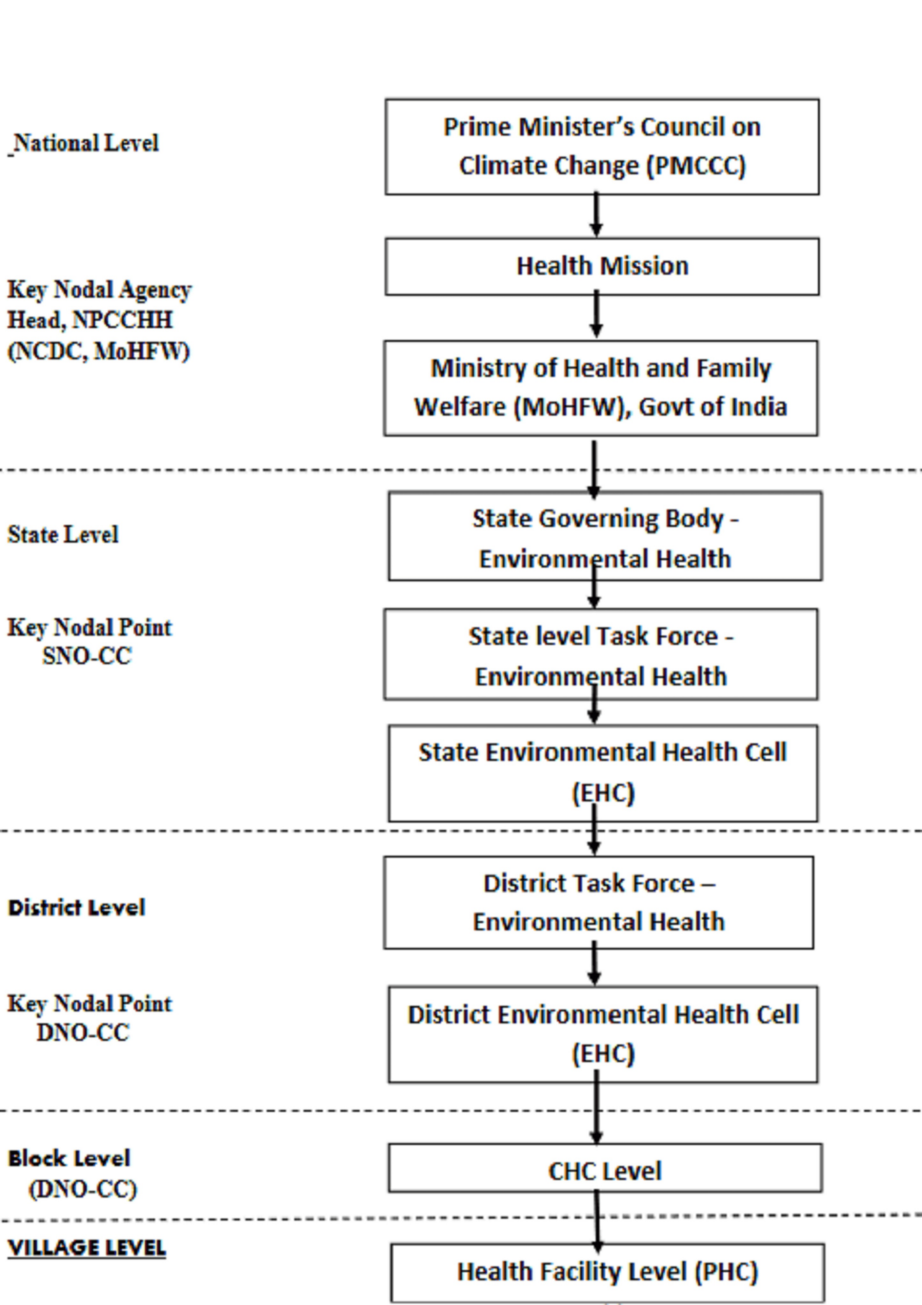
Organizational framework for national action plan for climate change and human health.

In 2008, the Prime Minister’s Council on Climate Change (PMCC) in India formulated the National Action Plan on Climate Change. It included the eight following missions: the National Solar Mission, the National Mission for Enhanced Energy Efficiency, the National Mission on Sustainable Habitat, the National Water Mission, the National Mission for Sustaining the Himalayan Eco-system, a National Mission on Strategic Knowledge for Climate Change, a National Mission for a Green India, and a National Mission on Sustainable Agriculture [8]. Later, on January 19, 2015, it was extended with the following four new missions: Health, Waste to Energy Generation, Coastal Areas, and Wind. In July 2015, under the chairmanship of the Director General of the Indian Council of Medical Research (ICMR), the Government of India, a national expert group on climate change was constituted. The Ministry of Health and Family Welfare was identified as a nodal agency. The National Centre for Disease Control (NCDC) was recognized as the nodal technical agency for the health mission that developed the National Action Plan for Climate Change and Human Health, released on October 23, 2018 [9,10]. The National Action Plan outlined five objectives with emphasis on creating awareness among the general population (vulnerable community), healthcare providers, and policymakers; strengthening the capacity of the healthcare system to reduce illnesses/diseases; strengthening health preparedness and response by carrying out the situational analysis at national/state/district/below district levels cement partnerships and establish close coordination with other missions; and uplift research capacity to bridge the evidence gap on the impact of climate change on human health. It was provisioned to formulate the State Action Plans for Climate Change and Human Health (SAPCCHH), and also the organizational framework from the national to village level was developed for the action plan implementation to translate the objectives on the ground in diverse with considerable variations in geo-climatic conditions (Figure 2) [11].

India has successfully developed policies, programs, action plans, intra- and inter-coordination mechanisms, and institutional frameworks from the national level to the village for climate change and human health [12]. It could be further strengthened by adopting decentralized planning through district-and block-wise action plans for climate change and human health. Delhi state has already developed a District Action Plan for Climate Change and Health (DAPCCH) that needs to be extended across India [13].

Health financing

Ensuring that the health systems are resilient to climate change necessitates sufficient resources and funding. The adverse impacts of climate change on public health result in increased healthcare costs. Building adaptability and resilience in the health system requires a sustainable budget. Annual adaptation costs for climate change in developing nations are approximately USD 70 billion and are expected to rise to USD 140-300 billion by 2030 and USD 280-500 billion in 2050, and India alone is estimated to spend 1.05 trillion USD by 2030 [14,15]. However, in the financial year (FY) 2021-22, it spent about USD 0.16 trillion on climate adaptation 2021-22, just over 5.5% of its GDP on climate adaptation [16]. As a result, governments must dedicate funds to strengthen the healthcare system's ability to withstand challenges and support initiatives for adapting to climate change. Governments could consider implementing novel collaborative and cross-sector financing approaches to achieve this goal.

The government's allocation of about 1.8% of the GDP towards public healthcare highlights a notable shortfall in funding [17]. This insufficiency poses obstacles to building climate-resilient health systems, as limited resources hinder the incorporation of advanced technologies, infrastructure enhancements, and sustainable practices. To bridge this gap, the government must open avenues for increased private investments to strengthen the health sector. It combines conventional budgetary assistance with creative financing methods like green bonds, climate funds, and blended finance.

Health workforce

The health workforce plays a crucial role in ensuring the resilience of the health system and providing essential health services. To achieve this, health workers require access to formal education and training that focuses on health, enabling them to diagnose, prevent, and manage health risks associated with climate change. Training should include an understanding of the impacts of climate change on the health system to enable the workforce to provide services during acute climate-related events. Additionally, efforts should be made to enhance the capacity of health workers to address health threats posed by climate change. It is essential to integrate climate-related illnesses and conditions into medical education at various levels, including undergraduate, postgraduate, and continuing medical education programs.

India's National Action Plan for Climate Change and Human Health emphasizes the need to train healthcare providers in technical skills, such as quality management, risk assessment, case management, epidemiology, disaster management, meteorology, monitoring and evaluation, and evidence generation [9]. In response to this, the Government of India, along with the state governments, have developed training programs for health adaptation and resilience to climate change, air pollution, and human health for women, children, traffic policemen, and municipality workers; prevention and management of heat-related illness for medical officers, community health workers, and communities; and nutritional disorders due to climate change for state and district nodal officers, medical officers, paramedical officers, and healthcare workers and communities [18,19]. The National Institute of Disaster Management (NIDM) also develops and organizes training programs like Climate Change Disaster Health: Epidemiology and Response Preparedness [20].

Despite the efforts made by governments at the union and states, the studies entail that the workforce has limited knowledge about climate change and health policies, particularly medical background professionals [21]. Studies also report a critical gap between formal education and training on climate change and health; hence, the curriculum for pre-service medical and allied health education needs to incorporate the competencies to deal with the new medical conditions that arise due to climate change [21,22].

Equipment and medicines

Sufficient facilities, equipment, and medicines are indispensable to ensure the provision of health services during acute climatic conditions. India is promoting telemedicine at healthcare facilities as part of the adaptation strategy. However, to strengthen telemedicine services, India needs to improve medical and technological infrastructure, urban-rural digital divide, technological illiteracy, lack of physical examinations, patient-provider relationships, appointment and consultation time, pool of experts, data security and privacy, and legal concerns [23].

Health information system (HIS)

HIS is essential for collecting, analyzing, and sharing data to aid managers in decision-making amidst the impacts of climate change. It is important to strengthen these systems to ensure that managers and health workers have access to necessary information on the vulnerability of the health system to climate risks, its capacity to respond, and its adaptability and resilience to the effects of climate change. This will enable evidence-based decision-making and enhance the health system's ability to effectively mitigate and manage the impact of climate-related health.

In India, there are two main Health Information Systems used for collecting and analyzing data to aid in decision-making for climate-sensitive illnesses/conditions at various administrative levels (including block, district, state, and country levels). These systems are the “Health Management Information System” (HMIS) and the “Integrated Health Information Platform” (IHIP). HMIS is a web-based Monitoring Information System that has been put in place by the Ministry of Health and Family Welfare (MoHFW), Government of India, to monitor the National Health Mission and other health programs and provide critical inputs for policy formulation and appropriate program interventions while IHIP aims to enhance the information accessible to both government health services and private healthcare providers regarding a specific group of critical diseases and risk factors and also to improve the on-the-ground responses to such diseases and risk factors [24,25].

Implementing HIS in India faces challenges in ensuring the completeness and correctness of data fields and using data at the point of care for improvement. Specifically, a study reports that healthcare workers face challenges due to interrupted networks, lack of reporting knowledge, and fear of government action [26].

Service delivery

Healthcare institutions need to have the necessary resources and capabilities to effectively address the impacts of climate change. This includes having the right facilities, medications, and equipment to deliver healthcare services during extreme climate conditions, tackling issues like heat stress, waterborne illnesses, as well as communicable and non-communicable diseases.

The NCDC has developed the Guidelines for Green and Climate Resilient Healthcare Facilities (2023) in which the specifics have been detailed as follows: how to make the health facilities energy efficient; how to conserve water; how to plan better the hospital building fire resistant, patient safety with comfort and how to make the hospital green by use of eco-friendly material, technology-based operations and how to better manage waste including general and biomedical waste [27]. Governments have made an implementation plan for climate-resilient health facilities, but it has yet to be implemented fully [28,29].

Conclusion

India has promptly initiated policy development to address climate change, including its impact on health. However, in order to effectively implement these strategies at the grassroots level, the healthcare systems require support through decentralized, bottom-up planning, adequate financial resources, and a robust data system. Additionally, efforts by both union and state governments to establish climate-resilient hospitals with competent staff need to be intensified in the coming days.
